# Psilocybin and ayahuasca in treatment-resistant depression: clinical evidence, methodological constraints and ethical challenges: a narrative review

**DOI:** 10.3389/fpsyt.2026.1858319

**Published:** 2026-09-15

**Authors:** Serena Chiara Civardi, Filippo Besana, Federico Durbano, Marco Otto Maria Toscano, Fabiola Raffone, Bernardo Maria Dell’Osso, Miriam Olivola

**Affiliations:** 1 Department of Mental Health and Addiction, Azienda Socio Sanitaria Territoriale (ASST) Melegnano e della Martesana, Vizzolo Predabissi, Italy; 2 Department of Mental Health and Addiction, Azienda Socio Sanitaria Territoriale (ASST) Fatebenefratelli-Sacco, Milan, Italy; 3 Department of Mental Health and Addiction, Azienda Socio Sanitaria Territoriale (ASST) Rhodense, Garbagnate Milanese, Italy; 4 Dipartimento di Salute Mentale e Fisica e Medicina Preventiva, Università degli Studi della Campania “Luigi Vanvitelli”, Naples, Italy; 5 Department of Mental Health, Azienda Sanitaria Locale (ASL) Napoli 1 Centro, Naples, Italy; 6 Department of Biomedical and Clinical Sciences “Luigi Sacco,” University of Milan, Milan, Italy; 7 Department of Psychiatry and Behavioral Sciences, Bipolar Disorders Clinic, Stanford University, Stanford, CA, United States; 8 ”Aldo Ravelli” Center for Neurotechnology and Brain Therapeutic, University of Milan, Milan, Italy

**Keywords:** ayahuasca, emerging treatments, psilocybin, psychedelic-assisted therapies, psychedelics, treatment-resistant depression

## Abstract

**Background:**

Treatment-resistant depression (TRD) is a major clinical challenge, with many patients failing to respond to conventional antidepressants. Psychedelic compounds such as psilocybin and ayahuasca have recently gained attention as potential rapid-acting therapies. Beyond their neurobiological effects, methodological, ethical, and real-world applicability concerns are critical for understanding their clinical utility.

**Methods:**

We conducted a narrative review of literature on psilocybin and ayahuasca in adult patients with TRD, focusing on safety, efficacy, and study design characteristics. PubMed and PsycINFO databases were searched from inception to August 2025. Studies were assessed for clinical outcomes, side effect profiles, and methodological and ethical limitations, including participant selection, blinding, and informed consent procedures.

**Results:**

Both psilocybin and ayahuasca demonstrate rapid reductions in depressive symptoms, with psilocybin showing more robust evidence for sustained effects. However, clinical trials are predominantly conducted in highly controlled environments with stringent inclusion and exclusion criteria, systematically excluding patients with comorbidities, high suicide risk, or polypharmacy. Challenges in maintaining blinding, high participant expectations, small sample sizes, and reliance on adjunct psychotherapy further limit the interpretation and generalizability of findings. Ethical considerations, including informed consent for intense psychedelic experiences and the potential for enduring personality or cognitive changes, remain under-addressed. Consequently, current evidence primarily reflects outcomes in clinically stable and selected subpopulations, rather than real-world TRD patients.

**Conclusion:**

Psychedelic-assisted therapies hold significant potential for TRD, but their current evidence base is constrained by methodological and ethical limitations. Future research should prioritize large, representative trials, standardized protocols, and robust safeguards to ensure both scientific validity and patient safety. Unlike previous reviews that primarily synthesized efficacy outcomes across different psychedelic compounds, the present narrative review specifically compares psilocybin and ayahuasca within the framework of treatment-resistant depression while integrating methodological, ethical and real-world implementation issues. This perspective aims at complementing the existing evidence by highlighting factors that may influence future clinical translation.

## 1 Introduction

Major Depressive Disorder (MDD) is one of the most prevalent psychiatric conditions (1), with 300 million people suffering from this disease worldwide. It also accounts for the largest proportion of disability-adjusted life years (DALYs) globally (2), leading not only to poorer health conditions and reduced life expectancy (3, 4) but also to limitation in quality of life (5).

MDD is a heterogeneous disease, encompassing a broad spectrum of symptoms arising from different environmental and neurobiological conditions. Given the complex and multifaceted nature of depression, it is not surprising that an important subset of patients does not achieve an adequate response to first-line treatments. Typically, conventional antidepressants need at least 4 weeks to demonstrate efficacy, with initial clinical improvement being evident after at least 2 weeks of treatment. A sustained response during these early phases of the disease is positively associated with an increased likelihood of remission (6, 7). On the contrary, a reduction in symptoms’ intensity of less than 20% after 2 to 4 weeks often requires a treatment adjustment (8).

Approximately 30% of MDD patients do not respond to standard pharmacological treatments. The Sequenced Treatment Alternatives to Relieve Depression (STAR*D) study, a large-scale clinical trial examining the response rate to conventional therapeutic approaches for depression, found that the cumulative remission rate after 4 trials with antidepressants within 14 months was 67% (9). Thase and Rush (10) distinguished between relative treatment resistance, treatment resistance, and refractoriness, classifying unresponsive patients along a continuum of five stages of resistance ranging from failure of one adequate trial of a single agent (stage I) to resistance to multiple classes of antidepressants and to bilateral electroconvulsive therapy (ECT; stage V) (11).

A consensus definition of Treatment-Resistant Depression (TRD) that is potentially meaningful in terms of clinical decision-making and health outcomes has not been reached yet. (12). The lack of a consensus definition leads to heterogeneity of patients enrolled in clinical trials, reducing interpretability and generalizability of the results.

Treatment-Resistant Depression is most commonly defined as an inadequate response to at least two different antidepressants administered at the correct dosage and for a suitable period of time (13). This definition corresponds to stage II of the Thase and Rush staging model for treatment resistance. The Thase and Rush staging model, despite factual advantages such as pragmatism and proximity to everyday clinical practice, lacks a consideration of depressive features like severity and duration of the index episode and does not take into account psychotherapeutic treatments.

At the state of the art, electroconvulsive therapy (ECT) remains one of the most effective interventions for TRD. However, this procedure is burdened by reduced accessibility and stigma (14). Therefore, novel therapeutic options have emerged. Among non-pharmacological treatments, neuromodulatory techniques such as repetitive transcranial magnetic stimulation (rTMS) have shown promising results (15). On the pharmacological side, esketamine, the levo enantiomer of Ketamine, has been tested for safety and tolerability in real world settings (16, 17) and has proven successful in improving TRD symptoms (18). Intranasal esketamine combined with a conventional antidepressant decreased the risk of relapse by 51% among those patients who had achieved stable remission and 70% among people who achieved a stable response with acute therapy (19). A recent review of real world data, involving more than 55000 patients, showed a clinically significant efficacy of Esktamine where administered together with both Selective Serotonin Reuptake Inhibitors (SSRI) or Selective Serotonin and Norepinephrine Inhibitors (SNRIs) in TRD patients (20).

In the light of the favorable outcomes of multiple clinical trials, esketamine has been approved by EMA and FDA as a treatment option for TRD (21, 22).

In the wake of the exploration of new rapid-acting antidepressants agents such as esketamine, a resurgence of research into psychedelics has begun. The term “psychedelic” comes from Ancient Greek ψυχή (soul) and and δηλοῦν, meaning ‘to reveal,’ ‘to manifest,’ or ‘to become visible’.

During the first half of the 20th century classical psychedelic agents, including psilocybin, mescaline and lysergic acid diethylamide (LSD) have generated substantial interest in both scientific and cultural domains. Aldous Huxley, for example, in *The Doors of Perception* (23), described the effects of mescaline as a “purification” of human perception, opening the way for a stream of para-scientific experiential literature. From the 1960s onwards, however, scientific research and also the use of psychedelics in psychiatric settings were halted for social and political reasons. The widespread recreational use of LSD within the “beat generation” and the broader youth counterculture led to strict restrictions. The Controlled Substances Act (1970, USA) and the UN Convention on Psychotropic Substances (1971) effectively enforced global prohibition (24–26).

Psychedelics are reemerging as treatment options for various psychiatric conditions. TRD is a field in which psychedelics are showing promising results, also in the wake of the Ketamine and Esketamine experience. Psychedelics have a unique mechanism of action that goes beyond the classic monoaminergic theory of conventional antidepressants and encompasses either glutamatergic, opioidergic, dopaminergic and serotoninergic systems eventually resulting in an increased brain plasticity (27).

In recent years, therapeutic potential of psychedelic substances has been investigated in the psychiatric community. Among them, psilocybin and ayahuasca have shown promising results in treating both MDD and TRD. In light of this, the aim of our narrative review is to summarize the existing literature dealing with the effects of these two molecules on TRD and to offer an overview of recent evidence.

Psilocybin is found in several mushrooms, particularly those of the Psilocybe genus. The first documented use of psilocybin dates back to the Aztec era. Aztecs knew it as “teonanacatl”, literally meaning “flesh of the gods”. During the 20th century ethnobotanist Richard Evans Schutes identified psilocybin species in Mexico and Robert Gordon Wasson introduced them to the western world after attending psilocybin ceremony with a Mazatec curandera (literally a traditional healer) (28, 29). The Swiss chemist Albert Hoffman ultimately synthesized psilocybin in 1958 from the Psilocybe Mexicana mushroom (30).

Psilocybin is a prodrug, therefore it becomes pharmacologically active once converted by alkaline phosphatase enzymes to psilocin, which is the active compound responsible for the psychoactive effects. Psilocin mainly acts as a partial agonist at the 5HT2A serotonergic receptor. Although with lower affinity, it also binds to 5HT1A and 5HT2B serotonergic receptors (31). Preclinical and clinical studies suggest that psilocybin may be associated with enhanced neuroplasticity, potentially via BDNF and mTOR-related pathways. However, these mechanisms remain putative and largely based on preclinical hypotheses inferred from biomarker and neuroimaging studies, without validation coming from human studies (27, 32).

Psilocybin psychoactive effects were characterized by Griffiths and colleagues (33). They found that psilocybin causes perceptual distortions, a sense of ego dissolution and union with the world (the so-called “oceanic boundlessness” feeling) and a wide range of intense emotions from panic to euphoria (34, 35). Somatic adverse events are generally mild and transient and include nausea, vomiting, and hypertension (36). A recent study on single dose administration to healthy subjects showed a dose-dependent side effects profile, but no critical concerns (37). Effects start 30 minutes after ingesting psilocybin, peak at 2 hours and last about 6 hours.

Beyond the acute psychedelic experience, psilocybin led to self-reported improvements in social interactions that lasted 2 months after administration (32). This finding suggests that it could potentially have long-lasting therapeutic benefits. As far as psychiatric diseases are concerned, psilocybin has shown promising potential in the treatment of obsessive-compulsive disorder (38), alcohol and substance use disorders (39, 40), anxiety and depression linked to terminal cancer (41, 42) and, above all, severe depressive disorder (43, 44).

Ayahuasca, literally “vine of the souls” or “vine of the dead” in the Quechua language, is a traditional Amazonian decoction with psychedelic properties. The use of ayahuasca dates back to the original inhabitants of the Amazonian region, where it was used by shamans for religious and healing purposes (45). During the 20th century, ayahuasca spread beyond indigenous contexts through Brazilian syncretic churches that combine Christian elements with African spiritualism and traditional indigenous religious practices.

The psychoactive brew is composed of the bark of the *Banisteriopsis caapi* vine, rich in beta-carboline alkaloids, mainly harmine, harmaline and tetrahydroharmine, and the leaves of the *Psychotria viridis* bush, that contain N,N-dimethyltryptamine (DMT). The beta-carboline alkaloids harmine and harmaline are monoamine-oxidase inhibitors (MAOIs) that enable DMT to reach systemic circulation without being inactivated by gut and liver MAOs. Tetrahydroharmine, on the other hand, acts as a weak serotonin reuptake inhibitor (46).

DMT is a serotoninergic psychedelic belonging to the same pharmacological class of psilocybin and acts as an agonist to serotonergic receptors 5HT2A, 5HT1A and 5HT2C with 5HT2A receptor being the main target. Preclinical and clinical evidence suggest that DMT may be associated with increased neuroplasticity, potentially through BDNF-related pathways (31). Fontanilla et al. (47) hypothesized that DMT is likely to serve as an endogenous sigma-1 receptor ligand. The function of this receptor is not well understood yet, but there is evidence that it may play a role in depression, anxiety, and cancer (48, 49). It has also been postulated that the activation of the sigma-1 receptor by DMT could hold potential in treating traumatic memories in post-traumatic stress disorder (PTSD) and dissociative amnesia (50). It is important to acknowledge that data on the sigma1 pathways are largely preclinical and clinical efficacy has not been demonstrated yet.

The ayahuasca experience usually starts about 40 minutes after ingestion, peaking between 1 and 2 hours. Effects last up to 6 hours. Ayahuasca effects include perceptual changes and alteration in consciousness, particularly depersonalization and derealization, together with an increased introspection and new insight into personal matters, leading to reinterpretation of hidden intrapsychic conflicts (49). The most common reported side effects are gastrointestinal malaise, nausea, vomiting and increase in heart rate and blood pressure. Adverse events are usually transient and non- life-threatening (51). In the field of psychiatry ayahuasca has provided evidence of effectiveness in ameliorating anxiety and depression symptoms (52).

Several recent reviews have summarized the antidepressant potential of psychedelic compounds in treatment-resistant depression. Among these, the systematic review by Horato et al. (53) comprehensively synthesized the available evidence regarding ketamine, esketamine and classical psychedelics, providing an important overview of efficacy and safety outcomes. Building upon this literature, the present review adopts a different perspective. Rather than providing another quantitative synthesis, we specifically compared psilocybin and ayahuasca in the context of TRD, emphasizing methodological limitations, ethical challenges, patient selection, psychotherapy integration and barriers to real-world implementation.

## 2 Materials and methods

This review follows a narrative methodology and does not adhere to PRISMA systematic review criteria. 15 studies have been included in the narrative synthesis. 12 of them investigate the role of psilocybin while 3 are focused on ayahuasca. All study designs were included. Among psilocybin studies, 9 have an open-label design and 3 are randomized clinical trials (RCTs). On the ayahuasca side, 2 studies are RCTs and 1 open-label.

### 2.1 Literature sources

For this narrative review, we adopted a structured search strategy. PubMed and Psycinfo databases were accessed to identify relevant research regarding the use of ayahuasca and psilocybin in treatment-resistant depression. “psilocybin TRD”, “psilocybin treatment resistant depression”, “ayahuasca TRD”, “ayahuasca treatment resistant depression” were used as free terms in both databases. The search dated back from the origin to April 2026. In order to ensure maximum thoroughness, a manual search was also performed to detect potentially relevant research that might have been missed during electronic search. Given the exploratory and hypothesis-generating nature of this review, no formal quality assessment or risk-of-bias tool was applied.

Although a structured search strategy was adopted, the choice of a narrative format lies on the heterogeneity of the included studies in terms of sample size, experimental design and clinical procedures. Also, due to the emergent nature of psychedelic therapies, we felt it appropriate to give extensive consideration to ethical and methodological considerations that are not easily analyzable in the context of the quantitative approach required by a systematic review.

The narrative design was deliberately to complement, rather than replicate, previous systematic reviews, particularly the work by Horato and colleagues, by allowing a broader discussion of methodological heterogeneity, ethical issues, clinical applicability and implementation challenges that cannot be adequately captured through qualitative evidence synthesis.

### 2.2 Eligibility criteria

We included studies investigating safety, efficacy and tolerability of both psilocybin and ayahuasca in adult subjects suffering from treatment-resistant depression. Studies unavailable in English, dealing with patients affected by MDD but not TRD, and lacking full text were excluded.

As far as the definition of TRD is concerned, all the included studies adopted the “classical” definition of TRD, i.e. failure to at least two adequate courses of antidepressant treatments administered in terms of both dosage and duration.

### 2.3 Screening and selection procedure

Research yielded 560 articles. Of these, 18 were deemed relevant and were included in the narrative review. Articles retrieved from the database search were at first screened independently by authors SCC and FB at title/abstract level to decide whether to proceed with the full-text screening. Secondly, full-text articles were assessed and data extraction was performed. Full text assessment was still carried out independently by SCC and FB. Any discrepancies both in title/abstract and full text screening procedures were solved by discussion with senior author MO.

### 2.4 Outcome measures

The selected articles were analyzed in terms of clinical and therapeutic outcomes, safety and tolerability, with particular reference to side effects and long-term impact of the treatment. The main features of the included papers are summarized in **Supplementary Material S1**.

## 3 Results

### 3.1 Comparative clinical evidence

#### 3.1.1 Psilocybin

Clinical outcomes of psilocybin-assisted therapy in treatment-resistant depression (TRD) have been evaluated primarily through open-label feasibility studies and randomized dose-controlled trials, using standardized and validated depression rating scales, including the Montgomery–Åsberg Depression Rating Scale (MADRS) (54), the Hamilton Depression Rating Scale (HAM-D) (55), the Beck Depression Inventory (BDI) (56), and the Quick Inventory of Depressive Symptomatology–Self Report (QIDS-SR16) (57).

Across studies, a consistent finding is the rapid onset of antidepressant effects, typically emerging within 24–72 hours following administration (58). Early open-label trials by Carhart-Harris et al. (58, 59), demonstrated marked reductions in depressive symptoms within the first week, with remission rates ranging from 58% to 67% in the acute phase and sustained response observed in approximately 40–50% of patients at 3–6 months. However, variability in long-term outcomes is notable, with some cohorts showing substantial attenuation of remission over time (59), highlighting inconsistencies in durability across studies.

Specifically, the two studies conducted by Carhart-Harris et al. in 2016 (58) and 2018 (59), conducted on 12 and 20 patients respectively. In both studies, participants received two dosing sessions consisting of a lower dose (10 mg) followed by a higher dose (25 mg) of psilocybin, administered within the same study protocol and combined with psychological support. In the first paper, significant reductions in depressive symptoms were observed within 24 hours after dosing, with maximal improvement occurring between Week 1 and Week 5. At Day 1 and Day 7, most participants met response criteria (≥50% symptom reduction), and remission rates ranged from 58% to 67% during the first month. At 3 and 6-month follow-up, approximately 40–50% of patients maintained clinical response, indicating moderate durability. According to standard criteria for determining remission (a score of ≤9 on the BDI), eight (67%) of the 12 patients achieved complete remission at 1 week and seven patients (58%) continued to meet criteria for response (50% reduction in BDI score relative to baseline) at 3 months, with five of these (42%) still in complete remission. Similarly, in another study (59) four patients (20%) maintained remission at week 5, three months and six months.

Neuroimaging-based open-label studies confirmed these findings. A study led by Carhart-Harris et al. (60), with a sample size of 20 subjects, reported response rates of approximately 45% at Week 5, while Daws et al. (61) observed sustained BDI improvements up to 6 months. These studies also demonstrated associations between symptom improvement and changes in large-scale brain network integration.

More recent open-label trials in specific populations further supported short-term efficacy. In an open-label pilot study with no control group, involving 15 U.S. veterans with TRD, Ellis et al. (62) reported large reductions in MADRS scores at Week 3 following a single 25 mg dose, with nearly half of participants maintaining response at Week 12. Similarly, Aaronson et al. (63) reported response rates exceeding 60% at Week 3, with partial maintenance at 12 weeks.

More robust evidence derives from the randomized, double-blind, dose-controlled trial by Goodwin et al. (64), which enrolled 233 participants with treatment-resistant major depression. Participants received a single administration of 25 mg (n = 79), 10 mg (n = 75), or 1 mg (control; n = 79) of psilocybin. These studies demonstrated a clear dose–response relationship, with significantly greater reductions in MADRS scores and higher remission rates in the 25 mg group compared with lower doses and control.

Specifically, at Week 3, the 25 mg group showed significantly greater reductions in MADRS scores compared with the control group, while the 10 mg group demonstrated intermediate effects. Remission rates (defined as MADRS ≤10) at Week 3 were 29.1% (23/79) in the 25 mg group, 17.3% (13/75) in the 10 mg group, and 7.6% (6/79) in the 1 mg control group, supporting a dose–response relationship.

For safety data, see section 3.5.

Suicidal ideation or behavior was reported in 6 participants (7.6%) in the 25 mg group, 3 participants (4.0%) in the 10 mg group, and 1 participant (1.3%) in the control group during the double-blind phase.

Follow-up studies (65, 66) showed improvements in anxiety, functioning, and quality of life, although between-group differences diminished by Week 12.

Overall, across studies, psilocybin-assisted therapy is associated with:

- Rapid symptom reduction (within 24–72 hours) (58, 64).
- Peak clinical response between Week 1 and Week 5 (59, 61).
- Remission rates of approximately 25–60% in the acute phase (58, 62, 64).
- Sustained response in 40–50% of patients at 3–6 months (59, 61).

#### 3.1.2 Ayahuasca

Clinical evidence for ayahuasca in TRD is more limited but includes placebo-controlled trials and longitudinal observational studies. Outcomes have mainly been assessed using MADRS and HAM-D scores (67, 68).

Across studies, ayahuasca is consistently associated with a rapid onset of antidepressant effects, typically emerging within 24–48 hours and peaking within the first week (67, 68). The most rigorous evidence derives from the double-blind, randomized, placebo-controlled trial by Palhano-Fontes et al. (68, n=35). In this study, patients with TRD received a single dose of ayahuasca (n=17) or placebo (n=18). Compared with placebo, ayahuasca produced significantly greater reductions in MADRS scores at Day 1, Day 2, and Day 7, with increasing effect sizes over time. At Day 7, remission rates were 36% in the ayahuasca group versus 7% in the placebo group.

Notably, Palhano-Fontes and colleagues reported an unusually high placebo response, with response rates of 46% at Day 1 and 26% at Day 7, substantially exceeding those observed in ketamine trials, in which placebo responses typically range between 0–6% at Day 1 and 0–11% at Day 7 (69–71). The authors attributed this elevated placebo effect to several contextual and clinical factors. First, participants were predominantly drawn from socioeconomically disadvantaged backgrounds and experienced significant psychosocial stressors. During the trial, patients were accommodated in a highly supportive and comfortable setting, which may have contributed to symptom improvement through enhanced therapeutic attention and care-related effects. Second, a high proportion of participants (approximately 76%) presented comorbid personality disorders, particularly within cluster B, a clinical characteristic that has been associated with increased placebo responsiveness (72). Together with the strong cultural and symbolic significance of ayahuasca, these factors may have amplified expectancy effects and contributed to symptom improvement in both treatment arms.

Open-label studies provided generally consistent results. Sanches et al. (67) observed significant reductions in both HAM-D and MADRS scores from Day 1 to Day 21 following a single ayahuasca session. Improvements were accompanied by reductions in suicidal ideation and anxiety symptoms. Neuroimaging analyses in the same cohort showed increased perfusion in limbic and paralimbic regions, including the subgenual cingulate cortex and nucleus accumbens.

Biomarker studies, such as Menezes Galvão et al. (74, n=28), documented acute modulation of cortisol levels and stress-related pathways, although direct correlations with clinical response were inconsistent. Menezes Galvão and colleagues observed that changes in cortisol level induced by ayahuasca did not correlate with improvement in depressive symptoms and this is probably due to the high heterogeneity of patients and the relatively small sample of volunteers. Indeed, authors acknowledged that the majority of patients presented comorbidity, such as personality disorder (76%) and anxiety disorder (31%).

Across available studies, ayahuasca treatment is associated with:

- Onset of antidepressant effects within 24–48 hours (67, 68).
- Peak improvement within the first week (68).
- Remission rates around 30–40% at Day 7 (68).
- Limited data beyond 3–4 weeks (67).

Taken together, these findings indicate that ayahuasca induces rapid and clinically meaningful short-term antidepressant effects, but that effect size, durability, and mechanisms of action remain highly context-dependent and insufficiently defined. From a clinical perspective, this raises important considerations. The apparent sensitivity of outcomes to set, setting, and patient characteristics suggests that translation into real-world practice may be particularly challenging, as such controlled environments are difficult to replicate outside research settings. Moreover, the limited longitudinal evidence and high placebo responsiveness call for caution in interpreting efficacy and highlight the need for larger, well-controlled trials with extended follow-up.

Overall, while ayahuasca represents a promising rapid-acting intervention for TRD, current evidence remains preliminary and does not yet support broad clinical implementation without further validation in ecologically valid, real-world populations.

To provide a clearer comparison between psilocybin and ayahuasca in the treatment of TRD, we have included a summary table (**Table 1**) highlighting key differences in study designs, clinical outcomes, onset and durability of antidepressant effects, side effect profiles, and underlying neurobiological mechanisms.

**Table 1:** Comparative evidence of psilocybin vs ayahuasca in treatment-resistant depression.

Feature	Psilocybin	Ayahuasca
Onset of antidepressant effect	24–72 hours (58, 59)	24–48 hours (68)
Peak clinical response	Week 1–5 (58, 59)	Within first week ( 67, 68)
Remission rates	67% at week 1; 29% at Week 3 (58)	36% at Day 7 vs 7% placebo (68)
Durability of effect	40–50% maintain response at 3–6 months in selected cohorts; attenuation by Week 12 in RCT (58)	Limited follow-up beyond 21–28 days; long-term durability largely unknown (67)
Somatic side effects	Most common were headache, nausea, dizziness, fatigue (64)	Nausea, vomiting, diarrhea, abdominal discomfort common (67, 68)
Psychological side effects	Suicidal behavior signal reported in high-dose arms in one phase II trial, paranoia, transient thought disorder; higher at high doses (64)	Transient emotional lability, dissociation, perceptual changes (in the first week) (67, 68)
Mechanistic pathways	5HT2A agonism, BDNF/mTOR neuroplasticity, large-scale network modulation (58, 59, 64)	5HT2A agonism (via DMT), MAOI-mediated DMT bioavailability, BDNF upregulation, limbic network modulation (67, 68, 73)

### 3.2 Clinical eligibility, external validity and therapeutic setting

#### 3.2.1 Study setting

Most psilocybin and ayahuasca studies were conducted in highly controlled clinical environments, typically university-affiliated research centers or specialized outpatient units (58, 59, 64, 68).

Interventions were delivered in day-hospital or outpatient settings, with continuous medical and psychological monitoring during dosing sessions. Participants generally remained under supervision for 6–8 hours after psilocybin administration (58, 64) and for approximately 4–6 hours following ayahuasca ingestion (67, 68). Overnight hospitalization was uncommon.

#### 3.2.2 Preparatory and integration procedures

All major psilocybin trials incorporated structured psychotherapeutic support, typically consisting of:

- Preparatory sessions focused on expectation management and therapeutic alliance
- One or two supervised dosing sessions
- Post-session integration meetings

Psychological support was delivered by trained psychiatrists, psychologists, or psychotherapists with specific experience in psychedelic-assisted therapy (58, 61).

Ayahuasca studies also included preparatory interviews and clinical follow-up, although integration protocols were more heterogeneous (61, 67, 68) and included supportive psychotherapies and mindfulness-inspired sessions.

#### 3.2.3 Inclusion and exclusion criteria

Across studies, patient selection was highly restrictive in order to minimize psychiatric and medical risks and to enhance internal validity (58, 59, 64, 68).

Common inclusion criteria were:

- Moderate-to-severe depressive symptom severity, typically MADRS ≥20 or HAM-D ≥17 at baseline (59, 68);
- Age between 18 and 65 years (58, 67).

The most frequent exclusion criteria included:

- Lifetime history of bipolar I disorder or psychotic disorders, and first-degree family history of schizophrenia or bipolar disorder (58, 59, 64);
- Current substance use disorder within the previous 12 months (58, 67);
- Active suicidal intent or recent suicide attempt (64, 68);
- Severe personality disorders, particularly borderline and antisocial personality disorder (58, 64);
- Significant cardiovascular, neurological, or hepatic disease (67, 68);
- Pregnancy or breastfeeding (59, 64).

In psilocybin trials, participants were required to discontinue serotonergic antidepressants, mood stabilizers, and antipsychotics prior to dosing, with washout periods ranging from 2 to 4 weeks (58, 59, 64).

In ayahuasca studies, additional exclusions concerned concomitant use of monoamine oxidase inhibitors, sympathomimetic agents, and medications associated with serotonergic toxicity or cardiovascular risk, reflecting the pharmacological profile of beta-carbolines (67, 68).

### 3.3 Psychotherapy and concomitant treatments

Most psilocybin trials required tapering and discontinuation of conventional antidepressants prior to dosing (58, 64). Psychotherapy was considered an integral component of treatment.

Ayahuasca studies were more heterogeneous: some allowed stable antidepressant regimens, while others required washout periods (67, 68).

Overall, outcomes may be influenced by the combined pharmacological and psychotherapeutic framework (59, 61).

Psychedelic-assisted psychotherapy (PAP) combines psychotherapy approaches with the use of psychedelic substances and is used mainly to treat Post-Traumatic Stress Disorder (PTSD) and TRD. The combination of psychotherapy with psychedelics can be conceived as a positive experience with long-term benefits in terms of mental health, rather than a mere neurochemical correction of brain malfunctions (74). PAP has a more direct impact on brain activity, which can result in profound insights and promote introspection, emotional release, and cognitive shifts (75).

The application of psychedelic-assisted psychotherapy in clinical contexts is not free from doubts and contradictions. Supporters emphasize the long-lasting positive effects while the skeptics focus on the scarcity of robust evidence and the lack of a defined set of guidelines due to the psychotherapy element involved. Indeed, both early and modern PAP models highlight the importance of non-directiveness and flexibility of the therapeutic framework and this is evident also from the integration of different psychotherapeutic approaches (e.g. supportive psychotherapy, cognitive-behavioral therapy, psychodynamic psychotherapy, existentially-oriented and mindfulness-based treatments) (76).

Long-term effectiveness of PAP and its superiority over classical treatments need to be investigated in prospective clinical trials. This will be useful also in designing specific training programs for future psychotherapists.

### 3.4 Pharmacokinetic and pharmacodynamic considerations

Differences in onset and durability of antidepressant effects across compounds appear aligned with their known pharmacokinetic profiles. Psilocybin, characterized by a longer subjective experience, is associated with symptom improvement emerging over days and peaking weeks later (58, 59, 64). In contrast, ayahuasca interventions, with rapid absorption and shorter acute effects, are consistently associated with earlier clinical response (67).

Pharmacodynamic studies converge on large-scale brain network modulation as a shared mechanism. Psilocybin studies consistently demonstrate decreased default mode network integrity and increased between-network integration (60, 61). Ayahuasca has been shown to increase regional cerebral blood flow in limbic and paralimbic regions (67), suggesting partially overlapping neurobiological pathways.

### 3.5 Safety and tolerability

Data on safety and tolerability of psilocybin and ayahuasca come from different sources ranging from systematic reviews to case reports. Given the heterogeneity of studies, caution is necessary in interpreting data in this field. Future research is needed, considering large samples and adopting rigorous double-blind procedures (74).

#### 3.5.1 Somatic adverse effects

A recent meta-analysis (36) found that psilocybin side effects, mainly consisting in headache, nausea, dizziness, fatigue and increased blood pressure, were mild to moderate in severity and never required medication for relief.

Ayahuasca studies uniformly reported nausea and vomiting as the most common side effects (67, 68), typically described as transient and expected. Less common physical adverse effects include abdominal pain, headache, chest pain and breathing difficulties. Moreover, a higher risk of physical adverse events was positively associated with older age at first ayahuasca use, a greater number of lifetime use, the presence of a physical health condition and a non-supervised context of ayahuasca use in comparison to religious setting.

#### 3.5.2 Psychological adverse effects

Overall, safety data are concordant in supporting acceptable tolerability under medical supervision, but divergence exists regarding the frequency of psychological adverse events, particularly in higher-dose psilocybin trials.

A systematic review by Gard et al. (77) highlighted that 5 out of the 17 cases of psychedelic-induced mania were due to psilocybin. Among these 5 cases, only one involved a subject without a history of bipolar disorder. Although the total number of psilocybin-induced manic episodes is reported to be low, the influence of psilocybin on people with bipolar disorder is still largely unexplored because these patients are typically excluded from clinical trials involving psilocybin as therapy. Weaker evidence comes from a case report (78), where the involved patient already suffered from bipolar II disorder.

There are also cases of psilocybin-induced psychosis (79, 80), paranoia (81) and transient thought disorder (82). Psychosis is more likely to develop after psilocybin use in those individuals consuming high doses of the drug or with predisposing factors (i.e. history of substance use, history of psychiatric symptoms or both). However, data are only available from case reports, so they cannot be generalized. The field of psilocybin-induced psychosis lacks large and prospective studies highlighting potential correlations between psilocybin’s psychedelic properties and the risk of developing psychosis both in individuals with known risk factors and in those without at-risk conditions. Particular attention should be paid to higher doses of psilocybin, as a recent study suggested a dose-response relationship (83).

Another concern that has been raised about the use of psilocybin is suicidality. In the recent phase II trial by Goodwin et al. (64) three participants, all suffering from TRD, experienced suicidal behavior. All three were known to be non-responders to psilocybin at the three-week endpoint and had previous suicide attempts or non-suicidal self-injurious behavior. Of note, all the three cases of suicidal behavior occurred in the high dose arm. Given the fact that TRD patients are particularly vulnerable, optimizing after-care following psilocybin treatment may be important to mitigate risks in critical populations. For example, another psilocybin-based trial conducted by Davis et al. (84) offered patients about 11 hours of supportive psychotherapy between preparation and one-month follow up (85).

As far as ayahuasca is concerned, some cases of manic symptoms have been reported after ingestion (86, 87). The former deals with a man with a known diagnosis of bipolar disorder, while the latter involves a woman who described a premorbid cyclothymic temperament but whose diagnosis of bipolar disorder followed ayahuasca consumption. Although fewer data are available compared to psilocybin, also in the case of ayahuasca, manic episodes are typically present in people suffering from bipolar disorder.

Ayahuasca use has also been linked to the development of psychotic symptoms. Data come from several case reports. In most cases, ayahuasca-induced psychosis has been described as short in duration and occurring in individuals with a personal or family history of psychiatric disorders or using other substances (88, 89). On the other hand, the case described by Baker and O’Shea (90) is about a man in his thirties with no personal nor family history of mental illnesses. Also, he had no history of substance abuse aside from occasional cannabis use and brief experimentations with stimulants at university.

Ayahuasca has also shown promising results in treating suicidality in TRD patients (91), that represent per se a particularly vulnerable population in terms of suicide risk (92–94). To date, there is only one case report reporting suicidal behavior following ayahuasca consumption. The person involved is a young man with a long history of depressive disorder who received ayahuasca in the context of a clinical trial (95). In this case, suicidal ideation was acute, transitory and not associated with a worse clinical outcome. Indeed, the patient completed the study and both the subjective report and the objective scales indicated that the treatment was effective on depressive symptoms. The authors acknowledge as limitation the lack of a structured assessment of suicidal ideation. In light of this, a suicide risk assessment should be performed when patients are administered psychedelics in clinical settings.

Generally speaking, all data of psychopathological sequelae following ayahuasca consumption come from case reports, so the evidence is weak and needs to be corroborated by large studies with adequate follow-up and structured assessment instruments.

In order to prevent, or at least reduce, the outbursts of the above mentioned psychopathological conditions, particular attention should be given to the context and setting of administration. Carhart Harris and colleagues (58) pointed out that the efficacy and safety of psychedelic therapies cannot disregard the context, both in the psychological and environmental sense. Various clinical trials have demonstrated that environmental and psychological support were crucial for successful psychedelic therapeutic sessions (24). More specifically, a special attention was paid to the creation of a pleasant environment, with low lighting, a selected playlist and aesthetically pleasing décor. An extensive and accurate psychological preparation was provided, with numerous visits before and after the psychedelic experience and a supportive-oriented psychotherapy. Moreover, patients were deeply connected with the dedicated mental health professionals. This way clinicians were able to carefully prepare the patients for their upcoming experience, provide them with support before and after it and help them integrate its meaning into their personal situations (96).

### 3.6 Subjective experience and clinical outcomes

Several psilocybin studies reported associations between mystical-type experiences and antidepressant response. Roseman et al. (97) identified oceanic boundlessness as a stronger predictor of symptom reduction than perceptual alterations or anxiety-related experiences. Similarly, Goodwin et al. (66) found correlations between emotional breakthrough and clinical improvement at higher doses. In contrast, Ellis et al. (62) and Ellis et al. [(98) long-term follow-up] found no such associations, highlighting heterogeneity across populations and study contexts.

Thus, while there are some evidence that certain experiential qualities may enhance therapeutic response, findings remain heterogeneous, and the necessity of intense subjective experiences for clinical benefit remains unresolved.

### 3.7 Comparative critical synthesis and clinical implications

A cross-study comparison highlights substantial variability in clinical outcomes for both psilocybin and ayahuasca, with remission rates, durability of response, and effect sizes differing markedly across trials. For psilocybin, remission rates range broadly (approximately 25–60% in the acute phase), with longer-term outcomes (3–6 months) showing attenuation in a significant proportion of patients. In contrast, ayahuasca demonstrates more consistent short-term effects (remission rates around 30–40% after 1 week), but lacks robust longitudinal data, limiting conclusions about durability. These discrepancies are likely driven by multiple moderating factors, including study design (open-label vs randomized controlled trials), sample size, baseline clinical heterogeneity, and differences in dosing protocols and psychotherapeutic support.

Importantly, contextual variables appear to play a particularly prominent role in ayahuasca studies, where high placebo response rates and sensitivity to set and setting suggest that non-pharmacological factors may substantially influence outcomes. In both interventions, patient selection criteria (e.g., exclusion of severe comorbidities, requirement for medication washout) and the intensity of psychological support further contribute to variability, potentially limiting generalizability to real-world clinical populations.

From a clinical perspective, these divergences have significant implications. The wide range of remission rates raises questions about predictability of treatment response and highlights the need to identify reliable biomarkers or clinical predictors of efficacy. Moreover, the apparent dependence on highly controlled therapeutic environments suggests that effectiveness in routine clinical practice may be lower than observed in trials. For psilocybin, variability in long-term outcomes underscores the need for maintenance strategies or repeated dosing protocols, while for ayahuasca, the lack of long-term data and high context sensitivity warrant caution in clinical translation.

Overall, while both compounds demonstrate rapid antidepressant effects, the heterogeneity of findings reflects an early-stage evidence base in which efficacy is strongly moderated by methodological and contextual factors. Future research should prioritize head-to-head comparisons, standardized protocols, and the identification of moderators of response to better inform clinical decision-making and optimize patient selection.

## 4 Integrative discussion and translational perspectives

The recent systematic review by Horato et al. represents the most comprehensive synthesis currently available on psychedelic therapies for treatment-resistant depression and provides an important reference framework for the field. Rather than reproducing the same findings, our narrative review was conceived to complement the research work by Horato and colleagues by focusing on aspects that are less amenable to systematic quantitative synthesis, including comparative clinical interpretation, methodological heterogeneity, ethical implications, patient selection, psychotherapy integration and challenges related to real-world implementation.

Our narrative perspective aimed at taking together two psychedelic drugs, one of which, psilocybin has been more deeply investigated, while the other, ayahuasca, is still relatively unexplored in its therapeutic potential. We specifically focused on TRD because this psychopathological condition bears a great burden of suffering for patients and represents a major clinical challenge.

To our knowledge, no previous review has specifically focused on the comparative clinical positioning of psilocybin and ayahuasca exclusively in the field of TRD also simultaneously integrating efficacy findings with methodological quality, psychotherapy requirements, ethical considerations, patient selection and barriers to implementation in routine psychiatric care.

Our main findings are summarized as follows: 1) both psilocybin and ayahuasca hold great potential as therapeutic options for TRD. However, evidence for ayahuasca is less strongly supported compared to psilocybin; 2) side effects are generally transient and non-severe, but, as far as psilocybin is concerned, the frequency and impact of important psychological side effects, such as suicide ideation, is still debated; 3) ayahuasca and psilocybin may share neurobiological pathways implied in the antidepressant effect, resulting in large scale brain network modulation.

The discussion is structured as follows: 1) Comparison with other rapid-acting antidepressants – particularly ketamine/esketamine, focusing on mechanisms of action, neuroimaging findings, and safety profiles. 2) Methodological and ethical biases in psychedelic trials – covering research ethics, informed consent, therapeutic ethics, and challenges related to the psychedelic experience itself. 3) Eligibility criteria and real-world applicability – analyzing how stringent inclusion/exclusion criteria limit generalizability to broader TRD populations. 4) Future directions for clinical implementation – discussing strategies for safe, ethical, and effective integration of psychedelic therapies into community and clinical practice.

### 4.1 Comparison with other rapid-acting antidepressants

Our findings highlighted psilocybin and ayahuasca’s rapid-onset and clinically significant antidepressant effect in the peculiar population of TRD patients. Their mechanism of action features a direct modulation of serotonergic receptors, namely 5HT2A, together with promotion of neuroplasticity and the reorganization of brain networks.

Peculiar mechanisms of action and rapid-onset antidepressant activity are characteristics also of ketamine/esketamine, that are already approved for the treatment of depression. A systematic review (99) compared psilocybin and esketamine in terms of antidepressant activity, safety profile and sustained long-term effects. Authors found that esketamine showed rapid antidepressant activity and its effects, including reduction of depressive symptoms and suicidal thoughts, persisted up to 7 weeks. Psilocybin demonstrates both rapid and long-term antidepressive effects as well, with comparable effects to standard-of-care pharmacotherapy. Both esketamine and psilocybin have a favorable profile in terms of side effects, but due to mind-altering properties must be administered in controlled settings under rigorous medical supervision. Psilocybin can cause particularly intense and powerful alteration of consciousness, so therapeutic sessions must be held in the presence of a qualified therapist that supports the patient. Moreover, psilocybin treatment is typically offered together with psychotherapy (81). Another recent systematic review (100) compared psychedelics (both psilocybin and ayahuasca) and ketamine/esketamine in terms of biological mechanisms and associated neuroimaging and clinical changes. Psychedelics like psilocybin and ayahuasca directly target the default mode network, improving cognitive processes that often complicate depressive disorders. Ketamine/esketamine act on the default mode network as well, but in an indirect fashion, because they impact mainly on prefrontal areas (Affective Network, Reward Network and Central Executive Network). This specific action on prefrontal-striatal glutamatergic pathways may explain ketamine’s efficacy as an anti-anhedonic treatment and its pivotal role in increasing cognitive control over emotional stimuli, thus reducing negative affect mood stages.

To strengthen the clinical positioning of these interventions, **Table 2** summarizes key comparative features between psilocybin, ayahuasca, and esketamine.

**Table 2:** Comparative clinical positioning of psilocybin, ayahuasca, and esketamine in treatment-resistant depression.

Feature	Psilocybin	Ayahuasca	Esketamine
Onset of antidepressant effect	24–72 hours (58, 59)	24–48 hours (68)	Up to 24 hours (99)
Peak response	Week 1–5 (58, 59)	First week (67, 68)	Days to weeks (99, 100)
Duration of effect	Sustained in ~40–50% at 3–6 months (58)	Limited data beyond 3–4 weeks (67)	Up to 6–8 weeks with maintenance dosing (100)
Setting of administration	Highly controlled clinical setting with continuous supervision (58, 59, 64, 68)	Clinical research or supervised ceremonial settings (67, 68)	Certified specialized clinics (99, 100)
Suicidality signal	Mixed evidence; debated risk at high doses (58, 59, 64)	Very limited evidence; rare reports (67)	Documented reduction in suicidal ideation (99, 100)
Psychotherapy dependence	High (preparation, dosing support, integration) (58, 59, 64)	Moderate to high; heterogeneous protocols (67, 68)	Low to moderate; not mandatory (99, 100)

### 4.2 Methodological and ethical biases in psychedelic trials

Despite the encouraging results of various studies, the clinical use of psychedelics is not free from controversies for both ethical and clinical issues. A recent consensus statement tried to identify the main topics to consider: research ethics issues; managing expectations and informed consent; therapeutic ethics; training, education, and licensure of practitioners; equity and access; and appropriate role of gatekeeping. This work may be an important starting point to develop a wider consensus on ethical issues that can be the basis of solid grounded guidelines (101). Specific ethical challenges have been raised also regarding the psychedelic experience itself. Indeed, the core element of ego dissolution poses the question of the adequacy of informed consent, since the psychedelic experience is difficult to understand by psychedelic-naïve patients. Another key debate concerns whether personality changes—often reported in domains such as neuroticism, agreeableness, and openness—are transient or enduring (102). Some argue that this challenges the stability of personal identity (103), while others emphasize the inherently dynamic nature of identity itself (104). From the clinical perspective, some caution is needed in drawing definitive conclusions on the superiority of psychedelics’ efficacy over conventional treatments. A recent meta-analysis (105) has shown that subjects receiving control treatment in psilocybin trials achieve significantly less improvement in MADRS scores than participants receiving control treatment in SSRI or esketamine trials. More specifically, the control group had a standardized mean change of only 0.50 in the psilocybin trials versus 1.00–1.12 in the SSRI/esketamine trials. This result is consistent with waitlist controls rather than placebo controls. The difficulty in maintaining treatment blindness and the excessive expectations of participants in psilocybin trials therefore necessitate the development of innovative strategies in defining experimental designs. However, this finding must be interpreted very cautiously, considering the study design and above all the clinical peculiarities of the TRD population, that represents a critical subgroup among patients with depression for the severity of the disease and the poorer prognosis.

Moreover, the evidence regarding ayahuasca is even more limited than psilocybin because, to date, fewer studies have investigated its therapeutic properties and the number of treated patients is relatively small. Besides, unlike psilocybin, no study regarding ayahuasca has a considerable follow-up and this further limits the generalizability of results and the possibility to take a clear position about its beneficial effects.

Despite these limitations, these molecules differ fundamentally from conventional antidepressants or anxiolytics: they integrate pharmacological and non-pharmacological dimensions, earning them the designation of “molecules of complexity,” positioned at the intersection of biological, psychological, and social dynamics (106). At the same time, psychedelics seem to have a faster activity on mental processes, favoring a faster psychological work in assisted therapies, and new mechanisms of action still to be defined (107). While their diffusion must be carefully monitored to prevent misuse and commercial trivialization, psychedelics offer the opportunity to rethink the relationship between medicine, the individual, and society. Continued rigorous research will be crucial to defining their future role in clinical practice.

### 4.3 Eligibility criteria and real-world applicability

The exclusion criteria applied in the protocols were generally rigid and stringent. This substantially limits the external validity and generalizability of current findings. In routine outpatient and inpatient settings, individuals with treatment-resistant depression frequently present with multiple psychiatric and medical comorbidities, elevated suicide risk, clinically significant personality disorders, and complex polypharmacological regimens. However, these clinical characteristics—highly representative of “real-world” TRD—are systematically excluded from most psychedelic trials for safety and methodological reasons.

As a consequence, the available evidence primarily reflects outcomes in a highly selected, clinically stable, and relatively “clean” subgroup of patients, characterized by low comorbidity burden, limited pharmacological complexity, and reduced clinical risk. While this approach enhances internal validity, it substantially restricts the applicability of results to everyday clinical practice.

Therefore, current findings should be interpreted as relevant mainly to this restricted subpopulation and cannot yet be directly generalized to the broader, more complex TRD population commonly encountered in clinical settings. Further studies including more representative samples will be necessary to determine the real-world effectiveness, safety, and feasibility of psychedelic-assisted interventions in treatment-resistant depression.

On the contrary, since Esketamine treatment has been longer investigated, data are available on its use in “real world settings. In particular, the Italian “REAL ESK” study included a naturalistic and non-selected sample of TRD patients coming from different mental health facilities (16). These patients, compared to those usually included in esketamine studies, had a higher rate of psychiatric comorbidities (15% with personality disorders (PDs) and 6% with substance use disorders (SUDs) in the REAL ESK sample, both conditions represent exclusion criteria in most esketamine RCTs), longer disease duration (19 years), higher unemployment rates (48.3%) and more frequent add-on therapies (56.9% with mood stabilizers and 57.7% with antipsychotics) (16, 21, 108). However, reduction of depressive symptoms and remission rates were comparable to those reported in randomized-controlled trials. Psychiatric comorbidities are an important aspect to be taken into consideration also for future trials with psychedelics, since they are often responsible for treatment resistance in MDD patients (108, 109).

The “REAL ESK” study’s results of safety and efficacy of esketamine therapy have been further reconfirmed by a very recent systematic review and meta-analysis by de Oliveira Lapa and colleagues (110). This research work is particularly relevant as it systematizes evidence exclusively from real world studies for the first time.

### 4.4 Future directions for clinical implementation

The progressive translation of psychedelics from clinical trials to community clinics is not free from challenges. (111). First of all, inherent risks, both physical and psychological, must be minimized through accurate screening and clinical care (96). Another key issue is careful training for healthcare professionals who are involved in the psychedelic treatments. They must be trained to support acute altered states of consciousness and to guide patients through the critical phases of preparation and integration. The provision of psychedelic therapies in community settings may be undermined by shortage of mental health practitioners. Training and accreditation of a large cohort of psychedelic therapists will be essential to meet anticipated community demand for psychedelic-assisted therapies Non clinical issues that must be taken into consideration include adequate infrastructure. Modification of existing clinics will be required to render them fit for purpose. As far as perception and public opinion are concerned, it is essential to strike a balance between a narrative focused on dangers and an overly enthusiastic perspective that would lead to unreasonable immediate approval and prescription of psychedelic therapies. In this regard, regulation is mandatory to maximize benefits and minimize risks and harms, ensuring safety and ethical practice. From an operational point of view, it would be advisable to universally adopt a registry to collect outcome and safety data on the use of novel psychedelic therapies. An example of such a project is Psychedelic-Assisted Psychotherapy Research registry led by the Australian National University. Moreover, the duration of the follow-up period should be standardized and, above all, a dedicated follow-up must be always provided.

Anticipation of potential hurdles and difficulties is fundamental to keep research on psychedelics alive and to facilitate the progressive use of psychedelic therapies in clinical practice.

## 5 Conclusion

Psilocybin and ayahuasca are emerging as compelling therapeutic options for treatment-resistant depression, offering rapid and clinically meaningful antidepressant effects. Evidence indicates that psilocybin provides more robust and sustained benefits, whereas ayahuasca induces rapid improvements with limited long-term data. Both psychedelics modulate serotonergic signaling, enhance neuroplasticity, and influence large-scale brain network dynamics, distinguishing them mechanistically from conventional antidepressants. While clinical outcomes are promising, current evidence is constrained by small, highly selected samples, heterogeneous psychotherapeutic support, and stringent exclusion criteria, limiting generalizability to broader TRD populations. The safe and effective integration of these interventions requires careful patient screening, structured preparatory and integration procedures, and administration by trained professionals in controlled environments. The present review should be viewed as complementary to previous systematic evidence, particularly that provided by Horato et al. By integrating methodological, ethical and translational perspectives with current clinical evidence, we aim at promoting a more clinically oriented interpretation of psychedelic-assisted therapies and identify priorities for future research.

Future research should focus on large-scale, representative trials, elucidation of long-term efficacy, standardization of therapeutic protocols, and the establishment of ethical and regulatory frameworks to translate psychedelic-assisted therapies into routine clinical practice. With rigorous investigation and thoughtful implementation, psychedelics may represent a complementary option pending further high-quality evidence for individuals with treatment-resistant depression.
