# Supplementary material for: Psilocybin and ayahuasca in treatment-resistant depression: clinical evidence, methodological constraints and ethical challenges: a narrative review
Source: Front Psychiatry. 2026 Sep 15;17:1858319. doi: 10.3389/fpsyt.2026.1858319 (PMC13621360; doi:10.3389/fpsyt.2026.1858319)
Supplement: Supplementary file 1 [file Table1.docx]

### **Supplementary material**

**File S1**

| **Study** | **Study Design** | **Population** | **Mean age (years)**  **(M/F)** | **Dosage of the psychedelic substance** | **Adverse effects** | **Assessment** | **Results** |
| --- | --- | --- | --- | --- | --- | --- | --- |
| Carhart-Harris et al., 2016 | Open-label feasibility study, no control group | 12 patients with moderate-to-severe, unipolar, treatment-resistant depression | 42.67 Y  6M, 6F | **Psilocybin**  10 mg and 25 mg 7 days apart | Transient anxiety during drug onset (all patients), transient confusion or thought disorder (nine patients),  mild and transient nausea (four patients), transient headache (four patients)  there were  no reported cases of so-called flashbacks or persisting perceptual  changes. | Mini-International Neuropsychiatric Interview, the 21-item HAM-D, MADRS, and GAF; patient-rated scales (16-item QIDS, BDI original version, STAI-T, and SHAPS). Patients also received an electrocardiogram, routine blood tests, blood pressure,  heart rate, and physical examination.  Eligible patients attended a subsequent baseline functional MRI followed by an extensive preparatory session. Patients were invited to talk openly about their personal history (including thoughts on the origins of their depression), a discussion of psilocybin’s psychological effects, and simulation of aspects of the dosing session. Patients were contacted 1 day after  low-dose session to check on their wellbeing and monitor for any adverse events. Patients returned 1 day after their high-dose session for a post-treatment fMRI scan, then completed interim questionnaires (QIDS, STAI-T, and HAM-D). 1 week after high-dose session, all baseline questionnaires and assessments were repeated. Subsequent assessments (only QIDS) were done via email 2, 3, 5 weeks after the high-dose session. Final follow-up was done remotely at 3 months after the high-dose session, and included QIDS, BDI, STAI-T, SHAPS. | Interim questionnaires done the day after the high-dose session showed some reduction in depressive symptoms. QIDS depression scores were significantly reduced from baseline to 1 week and 3 months post-treatment, with the maximum effect at 2 weeks. BDI and clinician-administered ratings confirmed these results. All patients showed some reduction in depression severity at 1 week that was sustained in the majority for 3 months. According to standard criteria for determining remission (eg, a score of ≤9 on the BDI), eight (67%) of the 12 patients achieved complete remission at 1 week and seven patients (58%) continued to meet criteria for response (50% reduction in BDI score relative to baseline) at 3 months, with five of these (42%) still in complete remission. STAI-T anxiety scores were also significantly reduced at 1 week and 3 months post-treatment, as were SHAPS anhedonia scores for 1 week and 3 months post-treatment |
| Carhart-Harris et al., 2018 | Open-label feasibility study, no control group | 20 patients with moderate-to-severe, unipolar, treatment-resistant depression | 42.2 Y  14M, 6F | **Psilocybin**  10 mg and 25 mg 7 days apart | One patient became uncommunicative  during the peak of his 25-mg psilocybin experience but this normalised after the acute drug effects abated. Transient anxiety lasting for minutes (15 patients) and headaches lasting no more than 1–2 days (8 patients) were the most common side effects. 5 reported transient nausea but there were no cases of vomiting.  3 reported transient paranoia within the duration of the acute drug experience but this was short-lived in every case. There were  no reported cases of so-called flashbacks or persisting perceptual  changes. | Initial screening comprised of a psychiatric interview (MINI-  5) to confirm diagnosis, physical examination, routine blood  tests, ECG, urine test for drugs of abuse and pregnancy where  relevant, a breathalyser and the completion of baseline  assessments. Eligible patients medicated with an antidepressant were advised to stop this for the trial, to avoid suspected attenuation of psilocybin’s effects. They attended a pre-treatment MRI scan and psychological preparation visit. Patients were seen the day after the 10-mg session for debriefing and a post-treatment MRI scan, and for one final time 1 week after the 25-mg session. Subsequent follow-up measures were collected remotely. QIDS-SR16 ratings were collected at baseline and 1–3 and 5 weeks and 3  and 6 months post-treatment, with 5 weeks post-treatment regarded as the primary endpoint. BDI and STAI ratings were collected at 1 week and 3 and 6 months. SHAPS (anhedonia) was collected at 1 week and 3 months and HAM-D (depression, clinician-administered) and GAF (global functioning, clinician administered) ratings were collected at 1 week only. Patients performed the 11D-ASC at the end of each dosing day when the subjective effects of psilocybin subsided to a negligible level; ratings were done with reference to the period when effects were most intense. | Relative to baseline, QIDS-SR16 scores were significantly reduced at all six post treatment time points, with the maximum effect size at 5 weeks. BDI scores were significantly reduced at 1 week, 3 months and 6 months post-treatment; STAI-T scores were significantly reduced at 1 week, 3 months and 6 months post-treatment; SHAPS scores were significantly reduced at 1 week and 3 months post-treatment; HAM-D scores were significantly reduced at 1 week post-treatment; and GAF scores were significantly increased 1 week posttreatment. Suicidality scores on the QIDS-SR16 were significantly reduced 1 and 2 weeks post-treatment, with trend decreases at 3 and 5 weeks. Scores on the suicide item of the HAM-D were significantly decreased 1-week post-treatment. 11D-ASC factors experience of unity, spiritual experience and blissful state can be considered together and indicated as “USB”. Significant relationships were found between USB mean scores and insight during the 25-mg psylocibin experience and changes in QUIDS-SR16 scores at 5 weeks. |
| Carhart-Harris et al., 2017 | Open-label clinical trial | Patients with treatment resistant depression  16 patients considered for ASL analyses;  15 patients considered for BOLD analyses (mean age 42.8 years; 11M, 4F) | ALS analyses: 42.8Y 12M, 4F  BOLD analyses: 42.8 Y  11M, 4F | **Psilocybin**  10 mg and 25 mg 7 days apart | Not mentioned. | A revised version of the QIDS-SR16 for 24-hour measurement for the post-treatment scan was used. The following imaging and imaging-related procedures were also performed: anatomical scan; BOLD fMRI state acquisition; BOLD pre-processing; seed-based RSFC; resting state networks; integrity (within RSN RSFC); segregation (between-RSN RSFC) | For the patients included in the ASL analysis (minus one patient whose scan 1 rating was not collected), the mean depression score (QIDS-SR16) for the week prior to the pre-treatment scan was 16.9 ± 5.1, and for the day of the post-treatment scan, it was 8.8 ± 6.2. The mean QIDS-SR16 score at baseline was 18.9 ± 3, and for 5-weeks post-treatment, it was 10.9 ± 4.8. Mean change values for those included in the BOLD analyses were −7.3 ± 5.3 (change from scan 1 to scan 2) and −8.2 ± 5.2 (change from baseline to 5 weeks post-treatment). Six of the 15 (BOLD) and 16 (ASL) patients met criteria for treatment response (≤50% reductions  in QIDS-SR16 score) at 5 weeks. All but one patient showed some decrease in QIDS-SR16 score at week 5 (with one showing no change) and 47% met criteria for response. Whole-brain CBF was calculated pre and post treatment and contrasted . Only decreases in CBF were observed post treatment (vs pre), and these reached statistical significance in the left Heschl’s gyrus, left precentral  gyrus, left planum temporale, left superior temporal gyrus, left amygdala, right supramarginal gyrus and right  parietal operculum.Increased sgACC RSFC was observed with the posterior cingulate cortex/precuneous (PCC) post-treatment but this effect did not correlate with reductions in depressive symptoms between scan 1 and 2 and nor did it predict treatment response at 5 weeks. Increased vmPFC RSFC was observed with the bilateral inferior-lateral parietal cortex (ilPC) post-treatment. This effect did not correlate with reductions in depressive symptoms between scan 1 and 2 but did predict treatment response at 5 weeks, with responders showing significantly greater vmPFC-ilPC RSFC increases than non-responders. Decreased PH RSFC was observed with a PFC cluster incorporating the lateral and medial prefrontal cortex.  This effect did not correlate with reductions in depressive symptoms between scan 1 and 2 but did relate to treatment response at 5 weeks, with responders showing significantly greater PH-PFC RSFC decreases than non-responders. Amygdala RSFC was not significantly altered post treatment. Analyses of within network RSFC using 12 previously identified canonical RSNs revealed increased default-mode network, dorsal attention network, and  posterior opercular network RSFC post-treatment; however, these changes did not correlate with depression outcomes, e.g. the relationship between change in DMN RSFC and reduced QIDS-SR16 scores between scan 1 and 2 were non-significant and neither were changes in DMN RSFC predictive of outcomes at 5 weeks. Analyses of between network RSFC using the same 12 RSNs, revealed decreased RSFC between the DMN and right frontoparietal network and increased RSFC between the sensorimotor network and rFP; however, these effects  did not relate to reduced QID-16 scores between scan 1 and 2, nor response at 5 weeks. |
| Daws et al. 2022 | Single-arm,open-label clinical trial | 16 patients with TRD | 42.75 years  12M, 4F | **Psilocybin**  10 mg and 25 mg 7 days apart | Not mentioned | Patients attended a baseline clinical assessment and baseline eyes-closed resting-state fMRI. The follow-up fMRI and clinical assessment occurred 1 d after DD2. Patients attended an on-site clinical assessment at 1 week after DD2 and completed further clinical  assessment electronically at 3 and 6 months. BDI was measured at baseline and week 1, 3 months and 6 months after DD2. | Relative to baseline, significant  BDI reductions were observed at 1 week and still evident at 6 months. Brain network modularity was significantly reduced 1 d after psilocybin therapy This result implies a global increase in functional connectivity between the brain’s main intrinsic networks.  Pre-treatment versus post-treatment changes in modularity significantly correlated with change in BDI score at 6 months, relative to baseline These results imply that decreased brain modularity 1 d after psilocybin therapy relates to long-term improvements in depression symptom severity. Significant reductions in DMN network  recruitment and increased between-network integration between the DMN and EN and between the DMN and SN were observed 1 d after psilocybin therapy. Taken together, these findings indicate a clinically relevant decrease in brain network modularity following psilocybin therapy for TRD. |
| G.M. Goodwin et al. 2022 | phase 2 double-blind trial | 79 participants were in the 25-mg group, 75 in the 10-mg group, and  79 in the 1-mg group (control). | Psilocybin 25 mg: 40.2, 44F, 35M; Psilocybin 10 mg: 40.6, 41F 34M; Psilocybin 1 mg: 38.7, 36F, 43M; | Psilocybin, 25 mg-10mg-1mg | Adverse events occurred in 66 participants (84%) in the 25-mg group, 56 (75%) in the 10-mg group, and 57 (72%) in the 1-mg group. The most frequent adverse events reported in the 25-mg group with onset on the day of psilocybin administration (day 1) were headache (in 24% of the participants), nausea (in 22%), and dizziness and fatigue (in 6% each). From day 2 up to week 3 (primary end-point assessment), severe adverse events were reported by 9% of the participants in the 25-mg group, 7% of those in the 10-mg group, and 1% of those in the 1-mg group. The serious adverse events in the 25-mg group were suicidal ideation (in two participants) and intentional self-injury (nonsuicidal self-injurious behavior) (in two participants) and in the 10-mg group were suicidal ideation (in two participants), intentional self-injury (in one participant), and hospitalization (for severe depression, in one participant). No serious adverse events were reported from day 2 up to week 3 in the 1-mg group. | period of 3 to 6 weeks, during which antidepressants and other prohibited medications affecting the central nervous system were tapered and discontinued at least 2 weeks before the baseline visit (the day before psilocybin administration).  Participants were randomly assigned in a 1:1:1 ratio to receive a single dose of psilocybin of 25 mg, 10 mg, or 1 mg (control).. After at least 6 hours and when the psychedelic effects of the drug had fully dissipated, participants returned home.  The trial followed participants for 12 weeks after treatment. The MADRS was administered by experienced mental health clinician raters by telephone at baseline, on day 2, and at weeks 1, 3 (primary end-point assessment), 6, 9, and 12 | At baseline, depression was moderate (MADRS total score, 20 to 30) in 30% of the participants and severe (MADRS total score, ≥31) in 68% of the participants. Mean MADRS total scores at baseline were 31.9 in the 25-mg group, 33.0 in the 10-mg group, and 32.7 in the 1-mg group. A total of 6% of the participants had previous exposure to psilocybin. The least-squares mean change from baseline to week 3 in the MADRS total score was −12.0 points in the 25-mg group, −7.9 in the 10-mg group, and −5.4 in the 1-mg group. The difference in the least-squares mean change between the 25-mg group and the 1-mg group was −6.6 (95% confidence interval [CI], −10.2 to −2.9;The incidence of remission at week 3 was 29% in the 25-mg group, 9% in the 10-mg group, and 8% in the 1-mg group (odds ratio in the 25-mg group vs. the 1-mg group, 4.8 [95% CI, 1.8 to 12.8]; odds ratio in the 10-mg group vs. the 1-mg group, 1.2 [95% CI, 0.4 to 3.9]). The incidence of sustained response at week 12 was 20% in the 25-mg group, 5% in the 10-mg group, and 10% in the 1-mg group (odds ratio in the 25-mg group vs. the 1-mg group, 2.2 [95% CI, 0.9 to 5.4]; odds ratio in the 10-mg group vs. the 1-mg group, 0.7 [95% CI, 0.2 to 2.0]) |
| G.M. Goodwin et al. 2023 | Phase II, exploratory, fixed-dose, open-label study | 19 participants with TRD were dosed and completed the study | 42.2, 13F, 6M | psilocybin 25 mg | 17 TEAEs (treatment-emergent adverse event) were reported for 12 participants (63.2%). The most common TEAE reported was headache (six events; six participants [31.6%]) which were predominantly transient, reported as mild and moderate in five and one participants, respectively, and resolved on their own or with standard medication on the same day or day after. Three participants had a TEAE of blood pressure increased which were considered related or possibly related to study treatment, two were severe and treated with clonidine. One of these patients experienced chest heaviness and headache. All TEAEs resolved before the end of the study (Week 3), and none had a duration exceeding seven days. There were no reports of active suicidal ideation | The primary efficacy end point was the change in Montgomery-Åsberg Depression Rating Scale (MADRS) total score from Baseline (Day -1) to three weeks post-psilocybin administration (Week 3)    Key secondary end points included the proportion of participants with a response in MADRS total score from Baseline and remission at Week 3,  - change from Baseline in Clinical Global Impression–Severity (CGI-S) score at Week 3 and proportion of participants with a response based on clinical judgment    Exploratory end points included change in total score from Baseline to Week 3 in participant -EQ-5D-3 level (EQ-5D-3L) which captures an index of health state based on response to five dimensions of quality of life, -Generalized Anxiety Disorder 7-item (GAD-7),  - self-reported Quick Inventory of Depressive Symptomatology 16-item (QIDS-SR-16)  - the Clinical Global Impression-Improvement (CGI-I) - the change from Baseline to Day 2 in Positive and Negative Affect Schedule (PANAS)  -The acute subjective psychedelic experience was captured by the FiveDimensional Altered States of Consciousness questionnaire (5D-ASC) once the acute psychedelic effects had subsided (end of Day 1) [37–39]. The intensity of experience relating to each dimension of the 5D-ASC was captured on a VAS ranging from “No, not more than usually” (0 mm) to “Yes, much more than usually” (100 mm). | The mean MADRS total score at Week 3 was 16.8 which equated to a clinically meaningful mean change from Baseline of −14.9. This improvement was apparent at Day 2 and maintained throughout the three-week follow-up.    Response at Week 3 was evident in eight (42.1%) participants and was sustained throughout the follow-up period for 63.2% at Day 2, and 57.9% at Weeks 1 and 2. The same 42.1% of participants who were responders at Remission rates remained high throughout the follow-up period for 52.6% at Day 2, 47.4% at Week 1, and 42.1% at Week 2  The mean CGI-S score at Week 3 was 2.9 which equates to a −1.3 change from Baseline    Improvements in quality of life, wellbeing, anxiety, affect, and selfand clinician-reported depression were evident at all time points following psilocybin administration. Participants’ EQ-5D-3L mean total score at Baseline was 0.51 and improved by an average of 0.18 by Week 3. The EQ-VAS mean score at Baseline was 49.0 and improved by an average of 13.7 by Week 3. The GAD-7 mean total score at Baseline was 12.9 and decreased by an average of 4.1 by Week 3, which is considered a clinically meaningful improvement in anxiety symptoms |
| G.M. Goodwin et al. 2023 | Phase 2, double-blind trial (To characterise fully the nature of psilocybin’s antidepressant effects, we report findings regarding other important, exploratory efficacy endpoints of the Goodwin et al. (2022a) trial to further elucidate the role of psilocybin in the treatment of people with TRD) | 233 participants with TRD were randomised to receive a single dose of psilocybin 25 mg, 10 mg, or 1 mg (control)    *Same as “G.M. Goodwin et al. 2022” | Psilocybin, 25 mg: 40.2, 44F, 35M;  Psilocybin, 10 mg: 40.6, 41F 34M; Psilocybin, 1 mg: 38.7, 36F, 43M;    *Same as “G.M. Goodwin et al. 2022” | Psilocybin, 25 mg-10mg-1mg    *Same as “G.M. Goodwin et al. 2022” | Adverse events occurred in 66 participants (84%) in the 25-mg group, 56 (75%) in the 10-mg group, and 57 (72%) in the 1-mg group. The most frequent adverse events reported in the 25-mg group with onset on the day of psilocybin administration (day 1) were headache (in 24% of the participants), nausea (in 22%), and dizziness and fatigue (in 6% each). From day 2 up to week 3 (primary end-point assessment), severe adverse events were reported by 9% of the participants in the 25-mg group, 7% of those in the 10-mg group, and 1% of those in the 1-mg group. The serious adverse events in the 25-mg group were suicidal ideation (in two participants) and intentional self-injury (nonsuicidal self-injurious behavior) (in two participants) and in the 10-mg group were suicidal ideation (in two participants), intentional self-injury (in one participant), and hospitalization (for severe depression, in one participant). No serious adverse events were reported from day 2 up to week 3 in the 1-mg group.    *Same as “G.M. Goodwin et al. 2022 | Exploratory efficacy measures included the following participantrated measures:  -the Generalised Anxiety Disorder-7 item (GAD-7) which reflects anxiety severity  -the Quick Inventory of Depressive Symptomatology-16 item (QIDS-SR-16) , which gauges depression severity  -the Positive and Negative Affect Schedule (PANAS) , which assesses emotional states from a list of adjectives  - the Sheehan Disability Scale (SDS)  -Work and Social Adjustment Scale (WSAS), which are measures of function  -EuroQol–5-Dimensions 3-Levels (EQ-5D-3L), which assesses quality of life  -computer-based Digit Symbol Substitution Test (DSST) (Jaeger, 2018), a measure of global cognitive function incorporating executive function, processing speed, and attention | At Week 3, psilocybin 25 mg and 10 mg reduced total depression scores on the QIDS-SR-16. The difference in the LSM change from Baseline to Week 3 between the 25 mg group and 1 mg group was − 2.8, and the difference between the 10 mg group and 1 mg group was −Reduction in depression scores remained apparent at Week 12. The 25 mg dose was associated with an increase in the PANAS positive affect score and a decrease in the PANAS negative affect score at Week 3. For the PANAS positive affect total score, the difference in the LSM change from Baseline to Week 3 between the 25 mg group and 1 mg group was 6.2 and the difference between the 10 mg group and 1 mg group was 1.6 . For the PANAS negative affect total score, the difference in the LSM change from Baseline to Week 3 between the 25 mg group and 1 mg group was − 3.2 , and the difference between the 10 mg group and 1 mg group was − 1.6. The GAD-7 total score, a measure of anxiety, also demonstrated that the 25 mg dose produced greater improvement compared with the 1 mg dose than the 10 mg dose at Week 3. The difference in the LSM change from Baseline to Week 3 between the 25 mg group and 1 mg group was − 1.8, and the difference between the 10 mg group and 1 mg group was − 0.5 . The 25 mg and 10 mg doses also improved measures of function, including SDS total score as well as SDS lost days and unproductive days in the preceding week at Week 3 . The difference in the LSM change from Baseline to Week 3 between the 25 mg group and 1 mg group was − 6.5, and the difference between the 10 mg group and 1 mg group was − 4.0 . A similar improvement in function was observed in the WSAS total score. The difference in the LSM change from Baseline to Week 3 between the 25 mg group and 1 mg group was − 5.1, and the difference between the 10 mg group and 1 mg group was − 3.1 . Effects on quality of life (assessed by the EQ-5D-3L and EQ-VAS) and cognitive function (assessed with the DSST total score) were smaller. For the EQ-5D-3L, the difference in the LSM change from Baseline to Week 3 between the 25 mg group and 1 mg group was 0.06, and the difference between the 10 mg group and 1 mg group was 0. Corresponding values for EQ-VAS were 6.8and 4.3, and for DSST total score were 1.5 and 0.5 . The treatment differences at Week 12 were less pronounced; however, the same dose-dependent trends in the LSM change from Baseline were observed. |
| Ellis et al. 2025  1 | open-label trial | 15 US Veterans with TRD | 43.2 Y  13M 2F | Psilocybin 25 mg single dose | -Seven participants : mild-to-moderate headaches during or immediately after dosing and resolved within 24 h.  -three participants: nausea during dosing  -Two participants: retroperistalsis (dry heaving) with symptoms resolving within 1–3 h.  -Two participants: mild back pain during dosing that resolved before the end of the day.  -Two participants: increased psychological distress during or immediately after dosing, with no increase in suicidal ideation or suicidal behavior.  -One participant: mild worsening of tinnitus symptoms that persisted after dosing.  -One participant had blood pressure outside of the acceptable range prior to dosing. This participant had no adverse cardiac events during or following dosing. | One day before the psilocybin dosing session, participants completed baseline assessments that consisted of HRSD, Montgomery-Åsberg Depression Rating Scale (MADRS), QIDS, C-SSRS, and Sheehan Disability Scale (SDS). Vital signs and urine were also collected for a urine drug screen and pregnancy test (for participants of childbearing potential).  At the end of the dosing day, the study team administered the structured self-rated Five-Dimensional Altered States of Consciousness rating scale (5D-ASC).  Participants met with their therapist team virtually the next day (Day 1) for a 90-min Integration session.. At this visit, the study team conducted clinical assessments (MADRS and C-SSRS) and participants completed questionnaires. Participants completed clinical assessments and questionnaires at Weeks 1, 2, 3, 6, 9, and 12 post-dosing and had at least two subsequent Integration sessions with their lead therapist in the follow-up period. | -MADRS scores decreased by an average of −23.00 from Baseline to Week 3 (SD = 11.34; 95 % CI [−28.96, −17.03]; *t* = −7.85; *p* < 0.001). The Cohen's d effect size of MADRS score at Baseline versus Week 3 was *d* = 2.23 (CI 95 % [0.88, 3.58]). At Week 3, 60 % (*n* = 9) of participants met response criteria and 53 % (*n* = 8) met remission criteria. At 12 weeks, 47 % of participants maintained a response (*n* = 7), and 40 % (*n* = 6) maintained remission. Treatment response was defined as ≥50 % reduction in the [MADRS](https://www.sciencedirect.com/topics/medicine-and-dentistry/montgomery-asberg-depression-rating-scale) score, and remission as MADRS score of ≤10.  - The average decrease in QIDS scores from Baseline to Week 3 was −12.10 ( *SD* = 5.28; 95 % CI [−14.89, −9.32]; *t* = −8.87; *p* < 0.001).  -There was a decrease in total SDS from Baseline to each study endpoint (*F*(3, 26) = 13.65, *p* < 0.0001). Total SDS scores were negatively associated with the random effect of age (*F*(1,4) = 11.30; *p* = 0.02) and race (F(3,4) = 7.85; *p* = 0.04).  -There was no significant correlation between the 5D-ASC score on Day 0 and change in MADRS at Week 3 (*R* = 0.41, *p* = 0.15) or Week 12 (*R* = −0.13, *p* = 0.66). None of the sub-scale dimensions of the 5D-ASC were significantly correlated with change in MADRS. |
| Ellis et al. 2025  2 | open-label trial | 10 US Veterans with TRD (from the sample of Ellis et al., 2025 1) | 40.1 Y  8M 2F | Psilocybin 25 mg single dose | see Ellis et., 2025 | The outcome of interest was change in depression severity using the clinician-rated measure Montgomery-Åsberg Depression Rating Scale (MADRS). From Baseline to Month 12 post-dosing.  Secondary measures included change in self-reported depression severity, disability, and anxiety from Baseline to Month 12, measured by the Quick Inventory of Depressive Symptoms (QIDS) and the Sheehan Disability Scale (SDS), respectively.  Acute psychedelic experience was measured at the end of the dosing using the structured Five-Dimensional Altered States of Consciousness rating scale (5D-ASC). Exploratory outcomes included the correlation between acute psychedelic experience (5D-ASC) and change in MADRS scores from Baseline to Month 6 and Month 12.  Safety and tolerability were evaluated by assessments of adverse events and suicidality (measured using the C-SSRS and assessed clinically by the study psychiatrist). The study psychiatrist met virtually with the participant during medication taper, before dosing, and at each follow-up visit to evaluate mood and possible adverse events. | The mean MADRS score at Baseline was 35.3 (+/− 7.3). At Month 6, 80 % of participants met response criteria (*n* = 8), and 50 % (*n* = 5) met remission criteria. At Month 12, 40 % (*n* = 4) met response criteria, and 30 % (*n* = 3) met remission criteria. Across all study timepoints, total MADRS scores were significantly lower than Baseline (*F* (5,36) = 15.67, *p* < 0.0001). Significant decreases from Baseline were observed at Month 6 (*M* = −24.59), Month 9 (*M* = −20.84), and Month 12 (*M* = −16.71).  Total QIDS scores significantly decreased from Baseline to each study endpoint ( *F* (5,34) = 21.56, *p* < 0.0001).  There was a small decrease in total SDS score across all long-term follow-up study endpoints ( *F* (5,40) = 4.71, *p* = 0.002). A small increase in SDS score between Week 3 and Month 12 reached statistical significance ( *M* = 1.22, *SE* = 0.36, *p* = 0.03).  No significant correlation was found between 5D-ASC score on Day 0 and change in MADRS total score at Month 6 ( *R* = 0.03, *p* = 0.94) or Month 12 ( *R* = −0.04, *p* = 0.92). None of the sub-scale dimensions of the 5D-ASC were significantly correlated with either Month 6 or Month 12 change in MADRS score ( *p* > 0.05). |
| Fon et al., 2025 | open-label trial | 12 patients aged 18-65 with TRD | 40.6 Y  6M 6F | Psilocybin 25 mg single dose | On the day of dosing, two patients (16.7%) reported headache, rated as mild in severity in both cases.  Two patients had subacute onset of moderate insomnia, 1 week and 4.5 weeks after dosing. The former of these had additional onset of moderate psychomotor agitation 2 weeks after dosing.  One patient had worsening depressive symptoms with onset on the day of dosing, of moderate severity. | The primary outcome was change in clinician-rated Montgomery-Åsberg Depression Rating Scale (MADRS) score from baseline to 3 weeks post-dosing. Secondary endpoints included change in MADRS scores from baseline at follow-up visits 1 week and 12 weeks post-dosing, and percent of participants meeting response (MADRS ≤50% of baseline) and remission (MADRS score ≤10) criteria at each follow-up visit.  Secondary and self-reported outcome measures included the Quick Inventory of Depression Symptoms-Self Rating-16 (QIDS-SR-16), the Quality of Life Enjoyment and Satisfaction Questionnaire-Short Form (QLES-Q-SF) and the General Anxiety Disorder-7 (GAD-7) questionnaire. These scales were administered at baseline and subsequent visits. | There was a significant reduction in MADRS scores from baseline at the primary and secondary endpoints. At week 1, 75% of patients met both response and remission criteria. At Week 3 (primary endpoint), 66.7% and 41.7% of participants met response and remission criteria, respectively. Greater than 50% of patients met response criteria at all timepoints assessed with the exception of week 9. At week 9, 33% of patients met response criteria, all of whom also met remission criteria. At study endpoint (week 12) seven out of all 12 enrolled patients (58.3%) met response criteria and three patients (25%) were in remission.  QIDS-SR-16 scores were significantly reduced from baseline at week 1 and week 12.  There was a significant effect of time in the overall model, with significantly increased QLES-Q-SF scores from baseline at both post-dosing time points (week 3 and week 12). |
| Goodwin et al., 2025 | phase II, multi-center, randomized, fixed-dose, parallel group, double-blind, dose-finding clinical trial | 233 patients with TRD    *Same as “G.M. Goodwin et al. 2022 | Psilocybin, 25 mg: 40.2, 44F, 35M;  Psilocybin, 10 mg: 40.6, 41F 34M; Psilocybin, 1 mg: 38.7, 36F, 43M;    *Same as “G.M. Goodwin et al. 2022 | single dose of psilocybin 25 or 10 mg compared with 1 mg (control) | Adverse events occurred in 66 participants (84%) in the 25-mg group, 56 (75%) in the 10-mg group, and 57 (72%) in the 1-mg group. The most frequent adverse events reported in the 25-mg group with onset on the day of psilocybin administration (day 1) were headache (in 24% of the participants), nausea (in 22%), and dizziness and fatigue (in 6% each). From day 2 up to week 3 (primary end-point assessment), severe adverse events were reported by 9% of the participants in the 25-mg group, 7% of those in the 10-mg group, and 1% of those in the 1-mg group. The serious adverse events in the 25-mg group were suicidal ideation (in two participants) and intentional self-injury (nonsuicidal self-injurious behavior) (in two participants) and in the 10-mg group were suicidal ideation (in two participants), intentional self-injury (in one participant), and hospitalization (for severe depression, in one participant). No serious adverse events were reported from day 2 up to week 3 in the 1-mg group. | Depression severity was measured by the Montgomery-Åsberg Depression Rating Scale (MADRS). Psychedelic experience was evaluated using two measures: the 5D-ASC and Emotional Breakthrough Inventory (EBI). | Depression response correlated with select aspects of the psychedelic experience overall and for individual doses. At the 25 mg dose, 5D-ASC dimensions Oceanic Boundlessness (Pearson correlation coefficient *r* = −0.508) and Visual Restructuralization ( *r* = −0.516), and EBI (r = −0·637) were the variables with the strongest correlation to the Week 3 change from Baseline in MADRS score. |
| Roseman et al., 2018    sample from Carhart Harris 2016 and 2018 | Open-label feasibility study, no control group | 19 patients with TRD | 44.7 Y  12M 6F | **Psilocybin**  10 mg and 25 mg 7 days apart | The most common adverse events were transient anxiety (mostly mild) during drug onset (n=12), transient confusion or thought disorder (n=9), mild and transient nausea (n=4), and transient headache (n=4; [table 2](https://www.clinicalkey.com/tbl2) ). These adverse events were expected psychological effects of psilocybin. Subacute headache typically presented 1 day after the psilocybin session, and subsided after 1–2 days. Paranoia presented in only one patient, but this was mild and transient. No prolonged psychotic symptoms were observed in any of the patients. | The main clinical outcome for this analysis was the self-rated 16-item Quick Inventory of Depressive Symptoms (QIDS-SR16). Five weeks after the 25 mg psilocybin session was chosen as the primary endpoint.  secondary measures were QIDS-SR at 1-day, 1-week, 3-months, and 6-months; Beck Depression Inventory (BDI, original version) at 1-week, 3-months, and 6-months; Clinician rated Hamilton Depression Rating scale (HAM-D) at 1-week; Dysfunctional Attitudes Scale (DAS; measures trait pessimism) at 1-week and 3-months; Spielberger's Trait Anxiety Inventory (STAI) at 1-day, 1-week, 3-months, and 6-months; Life Orientation Test Revisited (LOT-R; measures optimism) at 1-week and 3-months; and Snaith-Hamilton Pleasure Scale (SHAPS; measures anhedonia) at 1-week and 3-months. Standard criteria for meaningful “response” were calculated for the depression rating scales (≥50% from baseline). The altered state of consciousness questionnaire (ASC) was used to measure the acute subjective experience. It was completed retrospectively by the patient as the psilocybin session was coming to an end (i.e., ~5–6 h post ingestion). The 5 dimensions are: OBN (Oceanic Boundlesness), AED (Anxious Ego Dissolution), *visionary restructuralization* (VRS), *auditory alterations* (AUA), and *vigilance reduction* (VIR). | OBN was a significantly better predictor of reductions in depression than both *VRS* and *AUA* (*z* = 1.64 and *z* = 2.01, respectively, *p* < 0.05) items that best relate to OBN correlate most strongly with positive clinical outcomes, while sensory phenomena correlate less, and anxiety is predictive of worse outcomes. |
| Palhano-Fontes et al. - 2019 | double-blind randomized placebo-controlled trial | 29 patients with treatment resistant depression | 42Y, Ayahuasca (N=14, 11F, 3M); Placebo (N=15, 10F, 10M) | Patients received a single dose of 1 ml/kg of placebo or ayahuasca  adjusted to contain 0.36 mg/kg of N, N-DMT | No serious adverse events were observed during or after dosing.  Although 100% of the patients reported feeling safe, the ayahuasca  session was not necessarily a pleasant experience. psychological distress. Most patients reported  nausea, and about 57% have vomited, although vomiting is traditionally not considered a side effect of ayahuasca, but rather part  of a purging process | MADRS and the HAM-D were used to assess depression severity. MADRS assessments were at baseline (one day before dosing), and at 1 (D1), 2 (D2), and 7 (D7) days after dosing. HAM-D was applied only at baseline and D7, as it was designed to access depression symptoms present in the  last week | MADRS scores were significantly lower in the ayahuasca group compared with placebo at D1 and D2, and at D7 . Between-group effect sizes increased from D1 to D7. Response rates were high for both groups at D1 and D2, and significantly higher in the ayahuasca group at D7 . Remission rate showed a trend toward significance at D7 (36% v. 7%,). HAM-D scores: Between-group effect size was large at D7 (Cohen's d = 0.98; 95% CI 0.21–1.75). Within-group effect size was large for the ayahuasca group (Cohen's d = 2.22;), and medium for the placebo group (Cohen's d = 0.46; 95% CI −0.27 to 1.18) |
| Menezes Galvão et al. 2018 | randomized double-blinded placebo-controlled trial  using a parallel arm design | 43 healthy  controls without history or  diagnosis of major illness or psychiatric disorders, and 28  patients with treatment resistant depression. Half of the patients and half of the controls  received ayahuasca while the other half received placebo | 35.3Y, Controls (N=43, 24F, 19M); Patients (N=28, 21F, 7M) | Volunteers  received a single dose of 1 ml/kg of ayahuasca (AYA) adjusted  to contain 0.36 mg/kg of N,N-DMT, or 1 ml/kg of placebo  (PLA) | not mentioned | The volunteers slept in the hospital. At 6:00 a.m. the  next day (D0) saliva samples were collected for measuring  awakening salivary cortisol  (response assessments consisted of 3 saliva samples collected at  (i) awakening (±5min), (ii) +30min, and (iii) +45min). Acute salivary cortisol changes (%) were assessed during dosing session at two instants: (i) immediately before dosing, and (ii) +1 h 40min after  the ingestion of placebo or ayahuasca.  On the following day (D1), volunteers slept again in the  hospital (admitted II). At 6:00 a.m. the next day (D2), 48 h  after dosing session.  MADRAS (D-1; D2) | patients in the ayahuasca group (n = 10) presented greater salivary cortisol  increases compared to the placebo group (n = 12). Controls of the ayahuasca group (n = 21) showed greater increases of salivary cortisol levels compared to the placebo group (n = 20). patients and controls that ingested ayahuasca, both showing similar changes of salivary cortisol at 1 h 40min after ingestion  of ayahuasca; patients median = 98.72, controls median=146.87;. Acute salivary  cortisol changes (%) observed during dosing session for patients were not significantly correlated with MADRS scores at D2.    No changes in AUC between D0 and D2, for within groups  and treatments were observed. At D2,  AUC of patients who ingested ayahuasca is similar to values of controls group who ingested ayahuasca. On the other  hand, patients that ingested placebo continued presenting lower  AUC.  For plasma cortisol no significant changes were observed between D0 and D2, within both groups. No statically significant correlations were observed between plasma cortisol and AUC of awakening salivary cortisol of D2  for patients nor controls, nor with MADRS scores in the patients  group. |
| Sanches et al., 2016 (including sample from Osorio et al., 2015) | open label | 17 patients with TRD | 42.7 Y  5M 12F | Single-dose ayahuasca (2.2 ml/kg) administered with inpatient support and monitoring | Vomiting, reported by 47% of the volunteers, was the only adverse effect recorded. Ayahuasca effects on thought content and sensory perception were considered mild and short-lived, and no dysphoric effects were reported. | Depressive symptoms were assessed with the Hamilton Depression Rating Scale (HAM-D) and the Montgomery–Åsberg Depression Rating Scale (MADRS), manic symptoms with the Young Mania Rating Scale (YMRS), general psychiatric symptoms with the Brief Psychiatric Rating Scale (BPRS), and dissociative symptoms with the Clinician Administered Dissociative States Scale (CADSS) . Scales were completed 10 minutes before ayahuasca administration (baseline) and 40, 80, 140, and 180 minutes after drug intake. The HAM-D, MADRS, and BPRS scales were also completed 1 (D1), 7 (D7), 14 (D14), and 21 (D21) days after ayahuasca administration.  The effects of ayahuasca on regional cerebral blood flow were assessed by single photon emission tomography (SPECT) imaging performed before drug intake and eight hours after treatment.Increased blood perfusion in the subgenual area, nucleus accumbens, and insula were observed after ayahuasca intake. Hypoactivation of these brain regions is usually associated with depression, while increased activation is usually associated with antidepressive effects. | HAMD scores significantly decreased from 80 min to day 21 (*P* < 0.01 at all time points). Baseline HAMD (SD) of 19.2 (5.5) decreased to 7.6 (4.7) by day 21. Secondary analysis demonstrated large reduction in suicidal thoughts in participants with baseline suicidality. |
